# Supplementary material for: Complex‐centric proteome profiling by SEC‐SWATH‐MS
Source: Mol Syst Biol. 2019 Jan 14;15(1):e8438. doi: 10.15252/msb.20188438 (PMC6346213; doi:10.15252/msb.20188438)
Supplement: Supplementary file 8 — Dataset EV7 [file MSB-15-e8438-s008.zip › feature_plots_string/P56381.pdf]

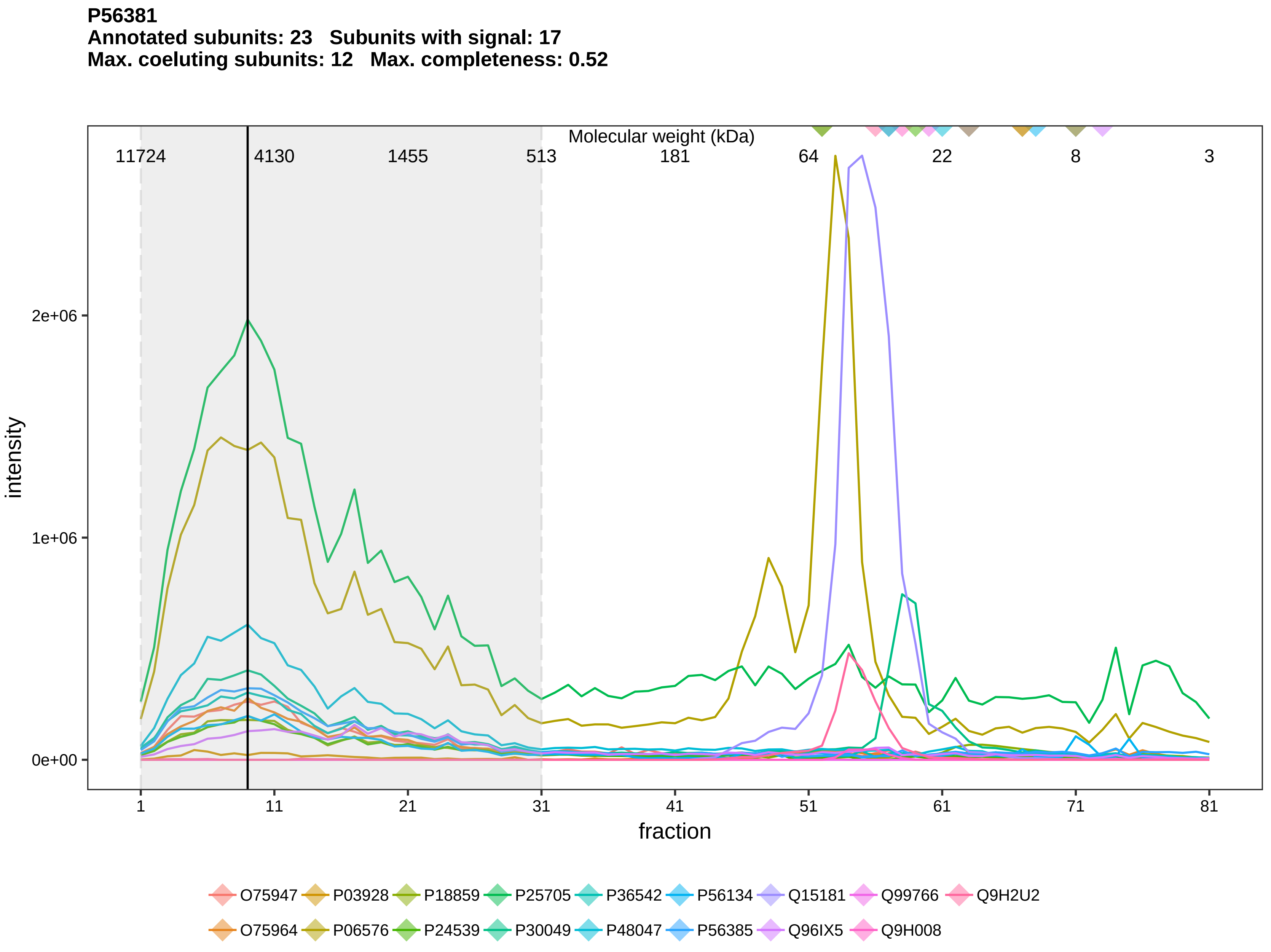

P56381
Annotated subunits: 23 Subunits with signal: 17
Max. coeluting subunits: 12 Max. completeness: 0.52
Molecular weight (kDa)
11724
4130
1455
513
181
64
22
8
3
intensity
2e+06
1e+06
0e+00
1
11
21
31
41
51
61
71
81
fraction
O75947
P03928
P18859
P25705
P36542
P56134
Q15181
Q99766
Q9H2U2
O75964
P06576
P24539
P30049
P48047
P56385
Q96IX5
Q9H008
